# GNL3L exhibits pro-tumor activities via NF-κB pathway as a poor prognostic factor in acute myeloid leukemia

**DOI:** 10.7150/jca.95339

**Published:** 2024-05-30

**Authors:** Ji Li, Zhimin Wu, Yipeng Pan, Yi Chen, Junfeng Chu, Yun Cong, Qingliang Fang

**Affiliations:** 1 Department of Hematology, the Second Xiangya Hospital, Central South University, Changsha, Hunan, 410011, China.; 2 Guiyang maternal and child health care hospital, Guiyang Children's Hospital, Guiyang, Guizhou, 550003, China.; 3 Department of Gastroenterology, Sir Run Run Shaw Hospital, Zhejiang University School of Medicine, Hangzhou, Zhejiang, 310020, China.; 4 Department of Breast and Thyroid Surgery, the Clinical Medical Research Center of Breast and Thyroid Tumor in Xinjiang, the Affiliated Cancer Hospital of Xinjiang Medical University, Urumqi, Xinjiang Uygur Autonomous Region, 830011, China.; 5 Department of Internal Medicine, Affiliated Cancer Hospital of Zhengzhou University & Henan Cancer Hospital, Zhengzhou, Henan, 450008, China.; 6 Department of Oncology II, Seventh People's Hospital of Shanghai University of Traditional Chinese Medicine, Shanghai, 200137, China.; 7 Radiation Oncology Department, Longhua Hospital, Shanghai University of Traditional Chinese Medicine, Shanghai, 200032, China.

**Keywords:** Acute myeloid leukemia, GNL3L, Prognostic factor, NF-κB

## Abstract

Acute myeloid leukemia (AML) is the leukemia with the worst prognosis, and current knowledge of AML pathogenesis and available therapies for AML remain limited. 40% of AML patients exhibit elevated nuclear factor kappa B (NF-κB) activity, which provides a compelling rationale for targeting the NF-κB pathway in AML. Guanine nucleotide-binding protein-like 3-like protein (GNL3L) is a recently identified pro-oncogene that promotes NF-κB activation in a variety of malignancies. For the first time, we comprehensively examined GNL3L expression in AML, reporting GNL3L as a poor prognostic factor in three independent AML cohorts. GNL3L enhanced RELA activity, activated NF-κB, promoted AML cell proliferation, resisted apoptosis, and encouraged cytarabine resistance in AML. In conclusion, these data suggest a role for GNL3L in the malignant process of AML and as a promising therapeutic target.

## Introduction

Acute myeloid leukemia (AML) is a heterogeneous type of hematologic malignancies caused by clonal expansion and differentiation arrest of myeloid progenitor cells. It is the most common and fatal acute leukemia in adults, especially in the elderly. The incidence of AML is 4.3 per 100,000 per year, and increases with age with the median age at diagnosis of 68 years 1. The prognosis of AML varies by subtype and decreases with age with an overall 5-year survival rate of less than 30% 2. Although intensive cytarabine-based chemotherapy 3 and allogeneic hematopoietic stem cell transplantation (alloHSCT) 4, as well as other emerging therapies such as chimeric antigen receptor T cell (CAR-T) 5, have greatly improved survival in AML, a significant proportion of patients do not benefit from them due to intolerance and resistance. The development in identification of cytogenetic abnormalities, mutations and dysregulations of oncogenes and onco-suppressor genes 6 has provided new insights into the pathogenesis of AML. The overexpression of certain genes, such as BAALC 7, ERG 8, MN1 9 and EVI1 10, which confer an advantage to leukemic cells to proliferate and self-renew, trigger leukemogenic events and show prognostic significance.

NF-κB pathway controls multiple biological processes, including cell survival, apoptosis, invasion, and hematopoiesis 11. NF-κB transcription factor family comprises RELA/p65, RELB, c-REL, NF-κB1 (p105/p50), and NF-κB2 (p100/p52), which either homodimerize or heterodimerize to form various NF-κB complexes, and heterodimer p65/p50 is the most prevalent active NF-κB complex in mammalian cells 12. NF-κB complex is physiologically inactive in the cytoplasm through interaction with NF-κB inhibitory proteins (IκBs). It can be activated via the traditional (canonical) and alternative (non-canonical) signaling pathways respectively, and then move to the nucleus and start the transcription of target genes. In the canonical activation pathway, several stimuli, such as pro-inflammatory cytokines, activate the IκB kinase (IKK) complex, which encourages IκB phosphorylated, poly-ubiquitinated, and degraded to free NF-κB dimer. In the non-canonical pathway, NF-κB inducing kinase is necessary for activating the p100/RelB complex as it phosphorylates and activates the IKK complex, which in turn causes the degradation of p100 to p52 and the subsequent nuclear translocation of the p52/RelB complex. Most human malignancies, including AML 13-15, have constitutive activation of NF-κB, which increases inflammation in the tumor microenvironment (TME) and promotes cell survival and resistance to therapy by activating anti-apoptotic genes. About 40% of AML patients exhibited increased activity of NF-κB by dysregulation of upstream regulators in its pathway 16, and the primary AML cells from 35% of AML patients highly expressed NF-κB receptor agonist genes, which correlated with reduced overall survival 17. It was further found that NF-κB signaling induced the release of cytokines that act as growth and survival factors for leukemic cells and mediated resistance of AML cells to cytarabine treatment 18. De novo pediatric AML patients with low levels of phosphorylation of the NF-κB subunit RelA were able to benefit from standard chemotherapy containing cytarabine in combination with the proteasome inhibitor bortezomib 19. Multiple oncogenes 20, 21 reduce acute leukemia sensitivity to cytarabine by activating NF-κB, and inhibition of the NF-κB pathway restores AML sensitivity to cytarabine 22.

GNL3L is a newly discovered, evolutionarily conserved, GTP-binding nucleolar protein, essential for ribosomal pre-rRNA processing 23. GNL3L can regulate cell cycle and affect proliferation and apoptosis. GNL3L binds and stabilizes TRF1 during mitosis to facilitate the transition of cells from metaphase to anaphase 24, and also functions as a nuclear-cytoplasmic transporter to promote the S phase transition by encouraging the binding of cyclin D1 and CDK4 25. Recently, GNL3L is identified as a potential pro-oncogenic factor as well. GNL3L blocks ubiquitination to stabilize MDM2, thereby inhibiting p53 function 26. GNL3L is highly expressed in numerous malignancies 27, such as esophageal cancer, lung cancer, and gastric cancer. It is also found that GNL3L contributes to the resistance of colorectal cancer to Irinotecan 28. The RELA subunit of NF-κB is up-regulated by GNL3L to boost NF-κB dependent transcriptional activity, and knockdown of RELA disrupts anti-apoptotic function of GNL3L, suggesting that GNL3L regulates NF-κB during the initiation and progression of tumors 29. However, the expression and function of GNL3L in leukemia are still unknown.

Here, we explored the expression and prognostic value of GNL3L in AML by combining public databases and our own collected samples, and found that GNL3L is highly expressed in AML and is a marker of poor prognosis in AML. Knockdown of GNL3L down-regulated RELA activity, and attenuated the malignant biological behavior of AML cells, including proliferation inhibited, apoptosis promoted and sensitivity to cytarabine increased. This study identifies GNL3L as a novel regulatory molecule that activates the NF-κB pathway in AML to promote the malignant biological behavior of cells, providing a new strategy for the treatment of AML.

## Materials and methods

### Human tissue specimen

AML patients (n=78) diagnosed according to the 2008 revision of the World Health Organization (WHO) criteria and recruited at Henan Cancer Hospital between 2014 and 2015, as well as healthy controls (n=30), were included in this study (AML-HNCH), which was authorized by the Ethics Committee of Henan Cancer Hospital in accordance with the Declaration of Helsinki (Approval NO.: 2019122501). Leukemia cells of AML patients and peripheral blood mononuclear cells of healthy controls were obtained for further RNA and protein extraction, and prognostic information from AML patients was also collected to analyze the impact of GNL3L expression on prognosis.

### Cell culture and transfection

AML cell lines HEL and THP-1 were purchased from National Collection of Authenticated Cell Cultures (Chinese Academy of Sciences, Shanghai, China) and cultured in RPMI 1640 medium (Gibco, New York, USA) supplemented with 10% fetal bovine serum (FBS) and 1% penicillin-streptomycin at 37 °C and 5% CO2. Shanghai Gene Pharma (GenePharma, Shanghai, China) designed and synthesized the small interfering RNA (siRNA) used for knocking down GNL3L expression. Sense: 5'-CGAGGAAGGCUUAUUACAAGG-3' and antisense: 5'-UUGUAAUAAGCCUUCCUCGUG-3' were used as the siRNA-1 (#1) sequence, while sense: 5'-GCAGGACCAUUGAGAGCUACU-3' and antisense: 5'-UAGCUCUCAAUGGUCCUGCGU-3' were applied as the siRNA-2 (#2) sequence. Cells (1.5 ×10^5^/ml) were transfected with GNL3L-specific siRNA or control siRNA (siNC) using the Lipofectamine 3000 Transfection Reagent (Thermo Fisher, Massachusetts, USA). RT-qPCR and Western blot were conducted to verify the knockdown efficacy at 48h post-transfection.

### Cell proliferation assay

AML cell lines HEL and THP-1 as well as siRNA cells were inoculated into each well of 96-well plate at densities of 3,000 cells/well. CCK-8 kit (Dojindo Laboratories, Kumamoto, Japan) and serum-free RPMI 1640 medium were mixed at a ratio of 1:10 and 100 mL was added into each well 29. Optical density (OD) values were then determined with a Multiskan Absorbance Reader (Thermo Fisher, Massachusetts, USA) using a wavelength of 450 nm.

### Colony formation assay

Colony formation assay was performed as previously described 30. In brief, AML cell lines and corresponding siRNA cells were cultured in complete medium consisting of 0.8% methylcellulose (Stem Cell Technologies, Vancouver, Canada) and 1% FBS, which were incubated at 37°C for 14 days in a highly humidified atmosphere with 5% CO2. The colonies were stained with 0.4% crystal violet (Solarbio, Beijing, China) in methanol for 10 min at room temperature. Visible foci were tallied and analyzed.

### Flow cytometry analysis of cell apoptosis and cycle

AML cells (1×10^4^ cells/mL) were cultured into a 6-well plate for 72 h and then collected for apoptosis and cell cycle analysis respectively. For apoptosis, cells were resuspended in 300 mL of binding buffer with Apoptosis Detection Kit (BD Biosciences, Franklin Lakes, USA), then incubated with Annexin V-FITC (BD Biosciences, Franklin Lakes, USA) in the dark for 10 min, and finally, propidium iodide (PI) solution (BD Biosciences, Franklin Lakes, USA) was added just before flow cytometry analysis. For cell cycle analysis, cells were fixed in 70% cold ethanol and incubated with RNase (Solarbio, Beijing, China), and stained with PI, followed by flow cytometry analysis.

### 50% inhibitory concentration (IC50) of cytarabine against AML cells

Log phase AML cells (2×10^4^ cells/well) were seeded onto 96-well plates, and cytarabine (S1648, Selleck, Houston, USA) was administered at concentrations of 0, 1, 2, 4, 8, 16 and 32 μM with a final volume of 200 μL. Following incubation for 24 h at 37°C with 5% CO2, the effect of cytarabine on AML cells was also determined using CCK8 assay as described above. The half maximal inhibitory concentration (IC50) value was estimated from the OD value and was applied to evaluate the cytostatic efficacy of cytarabine against AML cells.

### RNA extraction and real time quantitative polymerase chain reaction (RT-qPCR)

RNA was retrieved from AML cell lines and human samples with Trizol reagent (Invitrogen, Waltham, USA), and then reverse transcribed with SYBR Green qPCR Master Mix for qRT-PCR (TaKaRa, Kusatsu, Japan). RT-PCR was performed according to the standard protocol 31 and the GNL3L-specific signal was amplified using the following primers: GNL3L forward: 5′ATGTGCGAATTCATGATGAAACTTAGACACAAAAATAAAAAGCC and GNL3L reverse: 5′CACCATGATATCCCGGATGAACTTGTCCAGGTAGAC. β-actin was amplified with the following primers: forward: 5′GGCGACGAGGCCCAGA and reverse: 5′CGATTTCCCGCTCGGC as an internal control to normalize the equal quantity of RT products used in PCR.

### Protein extraction and western blot

Proteins from AML cell lines and human samples were extracted using lysis buffer and measured with the BCA protein assay (Beyotime Biotechnology, Shanghai, China). Western blot was performed as previously described 32. In brief, 30μg of proteins were added in each lane and electrophoresed on 10% Sodium dodecyl-sulfate polyacrylamide gel electrophoresis (SDS-PAGE) (Beyotime Biotechnology, Shanghai, China). After electrophoresis, transfer, and blockage, the PVDF membranes (Thermo Fisher, Massachusetts, USA) were incubated with appropriate primary antibodies (anti-GNL3L (PA5-44941, Invitrogen, Waltham, USA), anti-β-actin (ab8226, Abcam, Branford, USA), anti-RELA (ab32536, Abcam, Branford, USA), anti-RELA (phospho S536) (ab76302, Abcam, Branford, USA), anti-Cyclin D1 (#55506, Cell Signaling Technology, Danvers, USA), anti-BCL-2 (#15071, Cell Signaling Technology, Danvers, USA), anti-BCL-XL (#2764, Cell Signaling Technology, Danvers, USA)) overnight at 4°C. Then, secondary antibodies anti-Rabbit (A0208, Beyotime Biotechnology, Beijing, China), and anti-Mouse (A0216, Beyotime Biotechnology, Beijing, China) were added respectively at room temperature for 1h and the immunoblots were marked using ECL (Thermo Fisher, Massachusetts, USA).

### Data collection and analysis from public databases

RNA-sequencing expression profiles and corresponding clinical information for 139 AML patients and 70 healthy controls were downloaded from the TCGA (https://portal.gdc.cancer.gov/) and GTEx (https://gtexportal.org/) respectively, which were performed as previously described 33, 34. In addition, the Series Matrix File of dataset GSE71014 containing RNA-sequencing expression and corresponding prognostic information of 104 AML patients was retrieved from GEO (https://www.ncbi.nlm.nih.gov/geo/). AML-TCGA samples were divided into high- and low-expression groups according to the median value of GNL3L mRNA expression. The “ggplot2” and “cluster Profiler” packages were used to perform Gene ontology (GO) and Kyoto Encyclopedia of Genes and Genomes (KEGG) enrichment analysis to explore the biological functions of GNL3L-related genes. Furthermore, Gene Set Enrichment Analysis (GSEA) was used to explore potential pathways for the top most enriched genes in the GNL3L high- and low- expression groups.

### Statistical analysis

All assays above were carried out at least in triplicate and all statistical analyses were performed using GraphPad Prism 8 (GraphPad, La Jolla, USA). Students' t-tests and one-way analyses of variance were used to compare means between and within groups. The data is shown as mean ± standard deviation. Results are presented as mean ± standard deviation. The Kaplan-Meier curves were constructed to estimate the survival outcomes. The importance threshold for assessing statistical significance was established at p < 0.05.

## Results

### GNL3L is a poor prognostic factor in AML and associated with active NF-κB pathway

**Figure 1:**
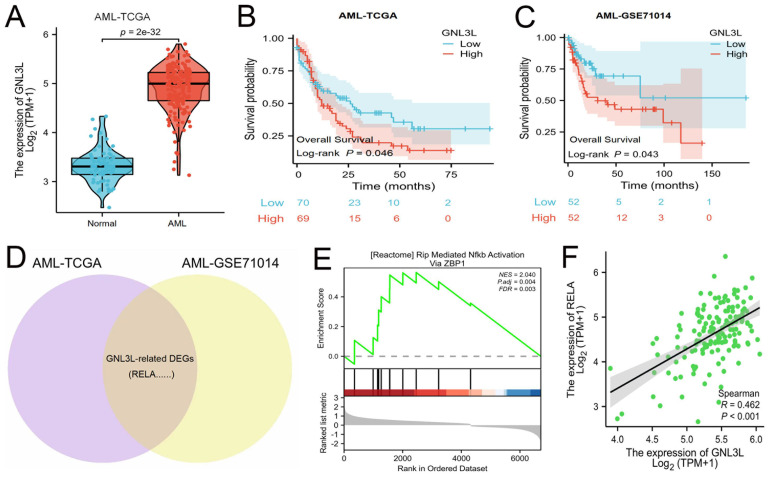
GNL3L is highly expressed in AML as a poor prognostic factor and associated with active NF-κB pathway. (A) The mRNA expression of GNL3L is up-regulated in AML in TCGA database. High expression of GNL3L predicts poor prognosis both in AML-TCGA database (B) and GSE71014 (C). (D) Venn chart of shared differentially expressed genes (DEGs) in AML-TCGA and GSE71014 dataset, including RELA. (E) GSEA shows NF-κB pathway is enriched in GNL3L high expression AML group. (F) The expression of GNL3L and RELA is significantly correlated in AML-TCGA.

The mRNA expression of GNL3L was first analyzed online in public databases of AML-TCGA and GSE71014, which showed GNL3L was up-regulated in AML patients compared with normal samples (Fig. 1A) and may play a role in AML. The median value of GNL3L expression was used as a cut-off score, and AML patients were divided into high and low GNL3L expression groups. Kaplan-Meier curves demonstrated overall survival of the high GNL3L expression group exhibited a significantly worse prognosis, suggesting GNL3L is a poor prognostic factor in AML (Fig. 1B, C). Differentially expressed genes (DEGs) were then identified to explore GNL3L associated genes and pathways with a total of 1,924 and 1,633 DEGs from AML-TCGA and GSE71014, respectively, according to the criteria of Log2FoldChange > 1 and p value < 0.05, and RELA was included in the 513 shared DEGs (Fig. 1D). In addition, the overlapped DEGs were subjected to functional enrichment analysis and GSEA was applied between the high- and low-GNL3L expression groups, which revealed Rip Mediated NF-κB Activation Via ZBP1 pathway (NES = 2.040, P. adj = 0.004, FDR = 0.003) was found to be significantly enriched in the high GNL3L expression group, suggesting that the high expression of GNL3L conferred the active NF-κB pathway in AML (Fig. 1E). Subsequently, the co-expression between GNL3L and NF-κB was explored in AML (Fig. 1F), and the expression of RELA was significantly positively correlated with GNL3L (R = 0.462, p < 0.001), indicating that RELA is a potential downstream target of GNL3L in the carcinogenesis and progression of AML.

### Validation of high expression of GNL3L and correlation with NF-κB in AML samples and cell lines

**Figure 2:**
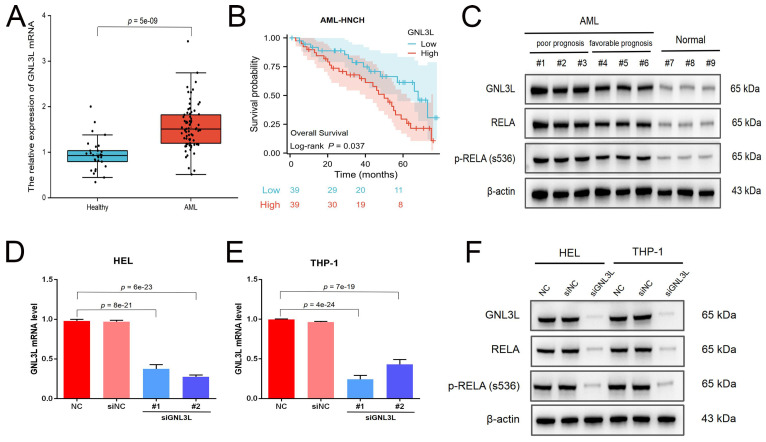
Validation of high expression and prognostic value of GNL3L in AML samples and cell lines. (A) The over-expression of GNL3L mRNA in AML was validated in the independent AML-HNCH cohort. (B) GNL3L high expression group exhibited poor overall survival in AML-HNCH. (C) The co-expression relationship between GNL3L and RELA was validated at the protein level in subgroups of AML-HNCH. siRNA knocked down the expression of GNL3L in HEL (D) and THP-1 (E) AML cells and RELA (F) as well.

The expression and prognostic value of GNL3L were validated in the independent cohort from AML-HNCH (Table 1). GNL3L was highly increased in AML both at the level of mRNA (Fig. 2A) and protein (Fig. 2C). AML patients were evenly divided into high and low GNL3L expression groups as above and high GNL3L expression group in AML-HNCH exhibited a prognosis as poor as in public databases (Fig. 2B). Meanwhile, the expression of RELA and its correlation with GNL3L were also validated in AML-HNCH. The expression and phosphorylation of RELA were significantly elevated in AML (Fig. 2C), and higher with a poorer prognosis, indicating that NF-κB was activated in the pathogenesis of AML caused by dysregulation of GNL3L as RELA plays the key role in the canonical activation of NF-κB pathway. Next, GNL3L knockdown cells were constructed by transfecting AML cell lines HEL and THP-1 with siRNA targeting GNL3L. Transfection efficiency was verified by RT-qPCR (Fig. 2D) and western blot (Fig. 2E), and the expression and phosphorylation of RELA were down-regulated by the knockdown of GNL3L, indicating that GNL3L participated in AML through RELA.

### GNL3L knockdown inhibited AML cells proliferation and induced G1/S cell cycle arrest

**Figure 3:**
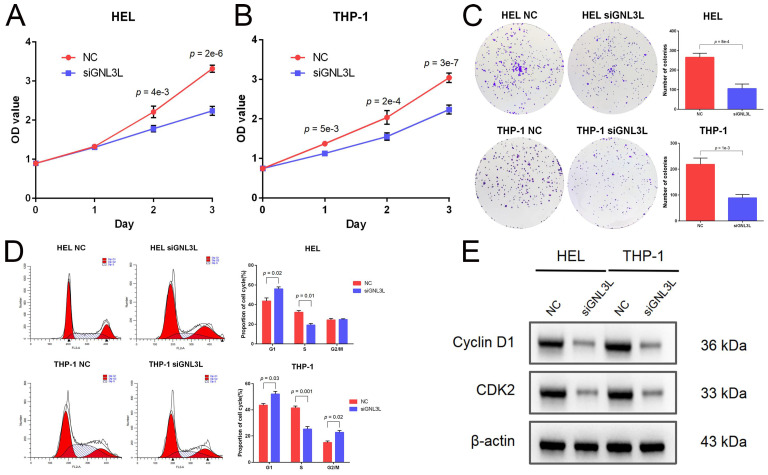
GNL3L knockdown inhibited AML cell proliferation and induced G1/S cell cycle arrest. (A) AML cells proliferated slower after knocking down GNL3L. (B) Number of colonies AML cells formed decreased after knocking down GNL3L. (C) AML cells were blocked at the G1/S cell cycle transition after GNL3L was knocked down. (D) Cyclin D1 and CDK2 were down-regulated in AML cells after knocking down GNL3L.

Cell viability of AML cell lines HEL and THP-1 was tested by CCK-8 assay after GNL3L knockdown, which showed GNL3L knockdown cells began to exhibit significant vitality impairment after 24 h or 48 h culture, and proliferated much slower (Fig. 3A). Colonies of AML cells also decreased after GNL3L knockdown (Fig. 3B), which suggested GNL3L regulates cell proliferation in AML. Flow cytometry was employed to examine whether GNL3L could affect cell cycle regulation and results showed that GNL3L knockdown induced G1 phase cell cycle arrest and reduced the fraction of cells in S phase (Fig. 3C). Furthermore, we also investigated whether the expression of cell cycle marker proteins was affected by GNL3L using western blot. The results indicated that GNL3L knockdown strongly reduced expression of cyclin D1 and CDK2 (Fig. 3D), which are important regulators of G1 to S phase transition.

### GNL3L knockdown induced apoptosis and reduced cytarabine-resistance in AML

**Figure 4:**
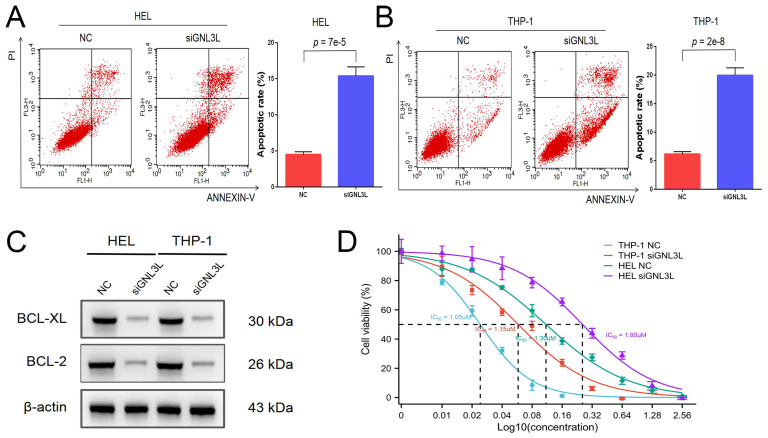
Knocking down GNL3L induced AML cell apoptosis and promoted sensitivity to cytarabine. (A) The apoptotic cells of AML cell lines HEL significantly increased after knocking down GNL3L, especially late apoptosis. (B) The apoptotic cells of AML cell lines THP-1 dramatically raised after knocking down GNL3L, mainly early apoptosis. (C) BCL-2 and BCL-XL were down-regulated in AML cells after knocking down GNL3L. (D) The sensitivity of AML cells to cytarabine increased after GNL3L knockdown.

AML cells were stained with Annexin V and PI and then apoptosis was determined by flow cytometry. After knockdown of GNL3L, HEL cells showed increased apoptosis, mainly late apoptosis (Fig. 4A), and THP-1 cells experienced an increase in apoptosis as well, primarily early apoptosis (Fig. 4B), suggesting that GNL3L can boost AML cell survival by resisting apoptosis. We next examined the effect of GNL3L on the expression of apoptosis-related markers using western blot. The results showed that after GNL3L knockdown the expression of anti-apoptotic proteins BCL-2 and BCL-XL were down-regulated (Fig. 4C), which are usually up-regulated by NF-κB pathway in other various cancers to promote survival, indicating that GNL3L exerts anti-apoptotic function in AML by regulating the expression of anti-apoptotic proteins BCL-2 and BCL-XL. Cytarabine is the most commonly used drug in AML chemotherapy, and AML cells were treated with different concentrations of Cytarabine, and the IC50 based on calculation from cell viability represents the sensitivity of cells to cytarabine treatment. The IC50 of HEL and THP-1 cells was significantly reduced after GNL3L was knocked down (Fig. 4D), with the greatest reduction in HEL cells, from 1.9 μM to 1.3 μM, suggesting that GNL3L is involved in the response of AML cells to cytarabine.

## Discussion

In this study, we demonstrated that GNL3L expression is up-regulated in AML and patients with high GNL3L expression have a lower overall survival rate, and GNL3L is an independent prognostic factor in AML. Cellular experiments showed that GNL3L depletion reduces the malignant biological behaviors of AML cells, including proliferation, anti-apoptosis and chemo-resistance. Pathway analysis revealed that NF-κB is activated in GNL3L-high expressing AML and that RELA, a core subunit of NF-κB, is highly associated with GNL3L, and its expression and phosphorylation are reduced by GNL3L knockdown. These data imply that GNL3L participates significantly in AML via the NF-κB pathway.

NF-κB signaling plays a crucial role in AML carcinogenesis, which is responsible for the differentiation, survival, growth, and resistance of leukemic cells 12. NF-κB has been demonstrated to be constitutively active in CD34+ stem cells from M3, M4, and M5 AML patients and is essential for preserving the proliferation and survival of AML stem cells 35. NF-κB promotes stemness of AML cells by activating LIN28B, and NF-κB inhibition reduces cell proliferation via downregulation of LIN28B 36, as well as self-renewal of leukemic stem cells *in vitro*, suggesting that inhibition of NF-κB may be a potential strategy to eradicate leukemic stem cells to counteract resistance and relapse. Furthermore, the ability of abnormal NF-κB signaling to activate anti-apoptotic genes like BCL-2 and BCL-XL is linked to the resistance of AML cells to radiotherapy and chemotherapy 37. Acquired resistance to venetoclax in AML cell lines is dependent on NF-κB activation 38, and the expression of numerous genes associated with the NF-κB signaling pathway is also altered during the development of cytarabine resistance. Proteasome inhibitors that reduce the activity of NF-κB signaling pathway could induce apoptosis in cytarabine-resistant AML cells 39. Additionally, NF-κB facilitates establishing AML drug resistance by controlling P-gp-mediated expression of the MDR1 gene 40. Several studies have shown that overexpression of the p50/p65 of NF-κB complex in drug-resistant AML cell lines induces the expression of P-gp genes and IAP family genes, which suggests the importance of NF-κB pathway in promoting progression, chemo-resistance, and poor prognosis in AML.

In AML, aberrant constitutive activation of NF-κB is due to increased activation of upstream regulators of the NF-κB pathway 41, in addition to mutational genetic alterations affecting genes controlling NF-κB activity and signals released by inflammatory stimuli and TME. DPP4, IL-1RAP, IRAK1, TAK1, and BTK 42-44 have been identified to be overexpressed in primary AML cells. Pim1 45 controls the NF-κB pathway by phosphorylating RELA/p65 at Ser276. DPP4 is required for the stemness and survival of AML cells via NF-κB. Inhibition of these genes prevents NF-κB activation and suppresses AML growth by promoting apoptosis *in vitro* and *in vivo*. Tumor suppressor gene TRIM10 46 inhibits the activation of NF-κB signaling pathway by suppressing the expression of RELA, thereby halting AML progression.

In other tumors, GNL3L is a pro-oncogenic factor that up-regulates the RELA subunit of NF-κB, promotes NF-κB-dependent transcriptional activity, regulates the cell cycle to affect proliferation, resists apoptosis, and promotes drug resistance. In this study, we for the first time explored the pro-tumor activities of GNL3L in AML and found that GNL3L promotes RELA expression and phosphorylation in AML and is an upstream regulator of the NF-κB pathway. However, the study's limitation is its *in vitro* nature, which limits the direct applicability of the findings to *in vivo* conditions. Future studies should validate these mechanisms in animal models and clinical studies to evaluate the therapeutic effects of targeting the GNL3L/NF-κB axis in AML patients. Our study suggests that GNL3L promotes malignant biological behaviors of AML cells through the NF-κB pathway and may be a new potential target for leukemia therapy.

## Conclusion

Our study highlights the critical role of GNL3L in exacerbating AML, identifying it as a key prognostic marker and a driver of disease progression through NF-κB pathway activation. GNL3L's promotion of cell proliferation, resistance to apoptosis, and cytarabine therapy resistance marks it as a pivotal factor in AML's poor prognosis. These findings suggest targeting GNL3L could offer a new therapeutic avenue in AML treatment, potentially overcoming current challenges in managing the disease. Our research paves the way for future investigations into GNL3L-targeted therapies, aiming to improve AML patient outcomes significantly.

## Figures and Tables

**Table 1:** Correlation of GNL3L expression with clinical and laboratorial parameters in patients from AML-HNCH cohort.

Parameters	Total (n = 78)	GNNL3L Expression		*p* Value
		High (n = 39)	Low (n = 39)	
Gender: male/female	42/36	22/17	20/19	0.65
Age, median (range)	57 (14-86)	60 (18-86)	62 (14-84)	0.77
WBC 10^9^/L, median (range)	24 (15.2-233.5)	32 (15.2-233.5)	20.4 (18-217)	0.05
Hemoglobin g/L, median (range)	71 (22-155)	65 (22-145)	76 (25-155)	0.13
Platelets × 10^9^/L, median (range)	50 (12-450)	42 (12-417)	55 (19-450)	0.46
Median BM blasts %, (range)	62.0 (23.0-95.0)	75.0 (34.0-95.0)	52.3 (23.0-89.5)	0.06
Cytogenetic risk (%)				0.12
Favorable	20 (25.6)	6 (15.4)	14 (35.9)	
Intermediate/normal	32 (41.0)	18 (46.1)	14 (35.9)	
Poor	26 (33.4)	15 (38.5)	11 (28.2)	
FAB classifications, n (%)				0.57
M0	5 (6.4)	3 (7.7)	2 (5.1)	
M1	8 (10.3)	5 (12.8)	3 (7.7)	
M2	11 (14.1)	7 (18.0)	4 (10.3)	
M3	18 (23.1)	5 (12.8)	13 (33.3)	
M4	16 (20.5)	9 (23.0)	7 (18.0)	
M5	15 (19.2)	8 (20.5)	7 (18.0)	
M6	3 (3.8)	1 (2.6)	2 (5.0)	
M7	2 (2.6)	1 (2.6)	1 (2.6)	

WBC: blood cell; BM: bone marrow; FAB: French-American-British.
